# Highly Selective Hybrid InSe-Graphene for NO_2_ Gas Sensing with High Humidity Tolerance

**DOI:** 10.1021/acssensors.4c03521

**Published:** 2025-07-01

**Authors:** Jyayasi Sharma, Frank Güell, Mubdiul Islam Rizu, Dalal Fadil, Eduard Llobet

**Affiliations:** † MINOS, School of Engineering, 16777 Universitat Rovira i Virgili, Avda. Països Catalans 26, Tarragona 43007, Spain; ‡ IU-RESCAT, Research Institute in Sustainability, Climatic Change and Energy Transition, Universitat Rovira i Virgili, Joanot Martorell 15, Vila-seca 43480, Spain; § TecnATox - Centre for Environmental, Food and Toxicological Technology, Universitat Rovira i Virgili, Avda. Països Catalans 26, Tarragona 43007, Spain; ∥ Catalan Photonics for Energy (ENFOCAT), 16724 Universitat de Barcelona, Barcelona, Catalunya 08028, Spain

**Keywords:** InSe, graphene, liquid phase exfoliation, humidity, gas sensor

## Abstract

p-type pristine InSe, pristine graphene, and the corresponding hybrid InSe-graphene gas sensor that is highly selective to NO_2_ have been developed. These materials are produced at an environmentally friendly temperature of 35 °C by the Liquid Phase Exfoliation (LPE) technique. Then their deposition was performed on alumina transducers for achieving chemoresistive gas sensors. X-ray diffraction (XRD), field-emission scanning electron microscopy (FESEM), high-resolution transmission electron microscopy (HRTEM), photoluminescence (PL), and Raman spectroscopy were used to analyze the materials. The multilayered crystalline structure is revealed by HRTEM. Studies on gas-sensing properties showed that the response of the hybrid InSe-graphene sensor to 1 ppm of NO_2_ is three times higher than the one of the pristine graphene sensor, whereas the pristine InSe sensor was not responsive. While under dry conditions, the response to NO_2_ (1 ppb) was 3.41%, under humid conditions (RH 50%), the responsiveness was significantly increased to 6.16% and to 14.42% for sensors operated at 150 and 250 °C, respectively.

## Graphical abstract

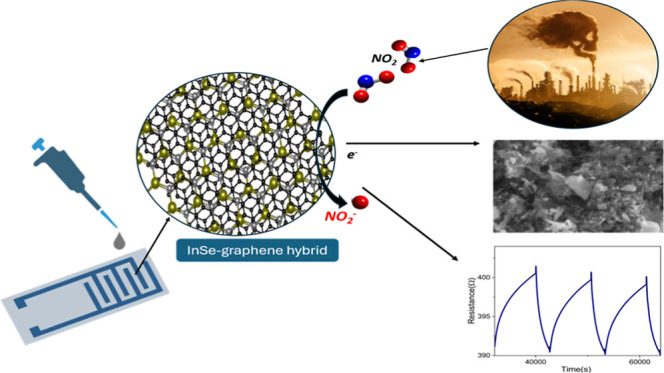

NO_2_ is a toxic gas that is reddish-brown and smells unpleasant.^1^ It primarily enters the environment from the combustion of fossil fuels.^2^ Acid rain and smog are two extremely damaging environmental phenomena that result from abrupt increases in the level of NO_2_ in the atmosphere.^3^ Long-term NO_2_ exposure can lead to several health problems, including skin conditions, respiratory tract malfunctions, asthma, and breathing difficulties.^4,5^ The World Health Organization (WHO) states that the recommended levels of NO_2_ for air quality control are 1 ppm, which should not be exceeded at any time. Additionally, 3 ppm is the limit for an 8 h work shift, and 5 ppm is the limit for very short-term exposure. Due to the growing danger of NO_2_ exposure, gas sensors for NO_2_ are crucial for monitoring our environment. Currently marketed affordable NO_2_ gas sensors typically have one or more significant flaws, such as poor selectivity, noisy response, slow response dynamics, low sensitivity at higher concentrations, high power consumption, and significant cross-sensitivity to ambient humidity.^6^

Metal oxides possess remarkable properties for developing inexpensive gas sensors, such as high sensitivity, low detection limits for many different toxic/explosive species, and simplicity (e.g., they are often implemented as chemoresistive devices). However, they have significant drawbacks in that they often operate at temperatures well above room temperature (e.g., 300 °C or even higher),^7–11^ which results in high power consumption. Additionally, they are characterized by a low selectivity with very significant ambient moisture cross-sensitivity.^12–15^ These characteristics make them unsuitable for widespread application. Thus, it is reasonable to conclude that the needs and specifications for industrial and environmental applications like portable devices installed in remote areas, identification of massive discharge of industrial waste gases, and monitoring NO_2_ levels in domestic households are not fully met by metal oxide gas sensors.^13,16–19^ Due to the limitations of metal oxides, 2-D materials have entered the gas-sensing and detection field. In that sense, graphene, MXenes,^20^ Black Phosphorus (BP), Transition Metal Dichalcogenides (TMDs), and Transition metal monochalcogenides (TMMs) have drawn a lot of interest.^21,22^ TMMs are composed of transition metals such as W, Mo, Ti, etc., and chalcogenides such as S, Se, Te, etc. Among these numerous TMMs, InSe is a (III–VI) layered compound whose distinct crystal structure and characteristics have drawn the attention of numerous researchers.^23–27^ InSe has a closely packed hexagonal crystal structure made up of Se–In–In–Se.^28^ There are three crystal forms of indium selenide: α, which has an indirect bandgap of 1.4 eV, β, which has a direct bandgap of 1.28 eV, and γ, which has a direct bandgap of 1.29 eV. At ambient temperature, the bulk bandgap of the β polymorph is 1.26 eV, while the monolayer bandgap is 2.11 eV.^29,30^ With its maximum interlayer and sliding energies, lowest Young’s modulus, and tunable bandgap, InSe becomes an excellent material for gas-sensing applications.^31^

For the preparation of materials such as InSe layers, common processes include chemical vapor deposition (CVD), Mechanical Exfoliation (ME), and Liquid Phase Exfoliation (LPE). However, CVD can only deposit films at high temperatures, and transfer of films onto their application substrate is required. ME has the drawback of creating nanosheets with small lateral diameters; its yield is low and thus presents scalability limitations.^32–35^ In contrast, there are several advantages to LPE, including low cost, ease of scalability, and yielding large area films/flakes.^36–39^ Some InSe-based gas sensors have been reported in the literature, but they have a few limitations. For example, they do not respond well to NO_2_ because of preadsorbed O_2_, and when there is ambient humidity present, signal recovery is slow and sluggish at room temperature, much like what has been observed in some other TMDs and TMMs. One of the major disadvantages experienced with pristine InSe sensors is that their high dependency on external environmental variables makes it difficult to guarantee sensor stability over an extended duration of time. Air exposure is primarily to blame for this deterioration. Apart from environmental instability, adverse effects such as thermal and photoinduced oxidation have also been documented.^40^ The chemisorption of O_2_ and H_2_O at Se vacancies is a crucial factor in the atmospheric oxidation of InSe.^41,42^ Due to the abundance of Se vacancies on the surface, oxidation begins there and continues throughout the sheet, replacing the Se atoms in the InSe structure with In–O bonds to produce the semiconducting In_2_O_3_.^42,43^

Graphene, which has a unique crystalline structure, is the most researched of the 2-D materials. It is available with sp^2^ hybridization in a single-layer honeycomb form.^44^ The unique properties of graphene, such as its optical qualities, mechanical strength, and thermal conductivity, are a result of its structure. An additional benefit of this sp^2^ hybridization for sensing performance (low noise) is the high mobility of charge carriers.^45–47^

Only a few InSe-based NO_2_ sensors have been reported in the literature previously. A pristine InSe sensor using gold interdigitated electrodes that detected NO_2_ under UV light excitation was described by Zhang et al.^48^ A combination of PdSe2/InSe for NO_2_ sensing that worked under UV light was described by Fan et al.^28^ Another combination of CTAB-intercalated InSe nanoscrolls that functioned under visible light excitation was reported by Zhang et al.^49^ InSe nanosheets for photoelectrical NO_2_ detection were also reported by Zheng et al.^27^ In–Se with oxides for NO detection was reported by Serra et al.,^50^ but this sensor was not responsive to NO_2_. However, when under humid environmental conditions, most of these sensors experience stability problems or their response degrades. To the best of our knowledge, no one has reported an InSe–graphene hybrid to detect gaseous species and, particularly, NO_2_. The hydrophobic properties of graphene may be of help for stabilizing InSe under ambient conditions, and for this reason, we explore the properties of hybrid InSe-graphene.

Herein, we report the development of pristine InSe, pristine graphene, and hybrid InSe-graphene sensors for the detection of NO_2_. The preparation method for these sensors is a simple 4 step process. (i) Solutions for exfoliation are prepared by mixing InSe in IPA solution for pristine InSe sensors, graphene in IPA solution for pristine graphene sensors, and InSe and graphene in equal amounts in IPA solution for hybrid sensors. (ii) Exfoliation of these three solutions by sonication. (iii) Centrifugation for the separation of sediments. (iv) Drop casting of the exfoliated nanosheets on alumina transducers. The sensing materials were characterized via FESEM, XRD, PL, HRTEM, and Raman spectroscopy to study the morphology, crystal structure, and orientation of the layers. We investigated the chemoresistive sensing mechanism of the pristine and hybrid sensors and studied the response of this combination to NO_2_ gas under dry and humid conditions. The hybrid InSe-graphene sensor displayed a 3 times higher response with respect to the pristine graphene sensor and showed high selectivity toward NO_2_.

## Experimental Section

### Preparation of Nanosheets

LPE was used to exfoliate the pristine graphene nanosheets. A solution of 2-propanol (IPA) (99.5%, Alfa Aesar) and DI water was added to 10 mg of graphene nanoplatelet aggregates (CAS-1034343-98-0) in a 3:7 ratio. Subsequently, the solution was sonicated for eight hours at 30 °C in a hot water bath to exfoliate pristine graphene nanosheets. Similarly, pristine InSe nanosheets were produced with 10 mg of InSe of bulk indium­(II) selenide powder (99.995%, CAS# 1312-42-1, Ossila) in 2-propanol (IPA) solution and the same exfoliation procedure was repeated. In the case of production of hybrid InSe-graphene nanoflakes, we mixed 5 mg of InSe and graphene, respectively, in IPA solution and repeated the same procedure of sonication. All three sonicated solutions were centrifuged for 30 min at 1500 rpm after exfoliation. The composite sediments were separated by size and thickness after centrifugation. The obtained sediments can be divided into 4 sections named T (the topmost), M (the middle), B (the bottommost), and precipitate. Each section T, M, and B comprises 2 mL of solution, and the precipitate comprises 1 mL of solution. The method of dividing the solution into 4 sections is crucial since LPE caused the created flakes to disperse in heterogeneous solution in terms of thickness and lateral dimensions. To obtain a large percentage of single-layered flakes, a purifying step is therefore required. This process is known as “Sedimentation-based-separation”.^36^

### Preparation of Gas Sensors

The solutions of pristine InSe exfoliated nanosheets, pristine graphene exfoliated nanosheets, and hybrid InSe-graphene exfoliated nanosheets divided into sections T, M, and B were deposited onto commercially available alumina substrates, featuring interdigitated platinum electrodes on the front side with a 300 μm electrode gap and platinum resistive meander on the back. The image of the alumina substrate can be found in the Supporting Information (see Figure S1). Each substrate was drop cast with 90 μL of solution, while being kept on a hot plate at 90 °C. Figure 1 illustrates the material and sensor preparation route.

**Figure 1:**
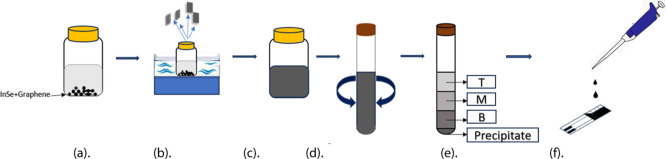
Schematic of the hybrid InSe-graphene sensor preparation method. (a) InSe + graphene mixed in IPA solution; (b) bath sonication of the solution; (c) exfoliated solution; (d) centrifugation of exfoliated solution; (e) separation of exfoliated solution for 4 different sections; (f) drop casting of solution onto alumina substrates.

### Physicochemical Characterizations

Field-Emission Scanning Electron Microscopy (FESEM)-Thermo Scientific Scios 2 was used to study the morphology of the sensing films. The Bruker AXS D8 diffractometer, which was outfitted with a vertical θ–θ goniometer, an XYZ motorized stage, a parallel incident beam (Gobel mirror), and a General Area Diffraction System (GADDS), was used to analyze the crystal structure using X-ray diffraction (XRD). Transmission electron microscopy (TEM) was done using ColdFEG (JEOL, Tokyo, Japan) that ran at 200 kV. The Raman spectra were obtained with a Renishaw inVia, laser 633 nm, argon-Novatech, 25 mW instrument. The PL measurements at RT were made using a chopped Kimmon IK Series He–Cd laser (325 nm and 40 mW). Fluorescence was dispersed with an Oriel Corner Stone 1/8 74000 monochromator, detected using a Hamamatsu H8259-02 with a socket assembly E717-500 photomultiplier, and amplified through a Stanford Research Systems SR830 DSP. A 360 nm filter was used to stray light. All spectra were corrected for the response function of the setups.

### Gas-Sensing Measurements

A 35 mL Teflon chamber with a homemade gas mixture and delivery system was used to test the sensors under different gas concentrations. The chamber accommodates up to 4 sensors. Using a Keysight BenchVue data collection system, sensing material resistance was monitored to acquire sensor response. Bronkhorst mass-flow controllers were used to combine calibrated cylinders of NO_2_, CO, and benzene with pure dry air. A constant flow rate of 100 mL/min was maintained. The analyte gas was exposed for 10 min, followed by a 30 min pure dry air recovery period. The humidity effect was investigated using a controller evaporator mixer from Bronkhorst High Tech. (Ruurlo, the Netherlands). The gas stream was humidified, and the humidity percentage was controlled by a Bronkhorst Flow View mass-flow controller. Sensors were exposed to dry air flow for at least 6 h before gas-sensing measurements commenced to ensure stable baseline resistances.

## Results and Discussion

### FESEM Analysis

FESEM was used for the morphological analysis of pristine InSe, pristine graphene, and hybrid InSe-graphene. The analysis revealed that pristine graphene comprises small-sized flakes ranging from 60 to 250 nm (edge to edge), while InSe has a multilayered structure. Pristine InSe nanosheets ranged in size from 460 to 800 nm, Figure 2a–c. The hybrids were obtained by mixing equal amounts of graphene and InSe (1:1 wt %). Sonication was done to evenly homogenize the mixture, as is evident in the FESEM images (Figure 2d–f). Furthermore, energy-dispersive X-ray (EDX) analysis was performed for elemental analysis of the hybrids. EDX spectra were taken at multiple locations of the sample in order to determine the average weight percentage of graphene in hybrid InSe-graphene, which was found to be 39% and indicated a C/O ratio of 1.5:1.^51^ Also, FESEM was used to characterize individual sections of the hybrid (T, M, and B) to analyze the sediments. The analysis revealed that heavy/larger flakes settle in section B and light/smaller in section T. It was observed that exfoliation improved with larger flakes and more defects as we moved from the top section T to the bottom section B, as shown in Figure 2d,e. This is clear evidence of enhanced exfoliation in the bottom section (i.e., section B). FESEM micrographs of nonexfoliated InSe and a chemical mapping (microanalysis) of exfoliated InSe/graphene flakes can be found in the Supporting Information (see Figures S2 and S3, respectively).

**Figure 2:**
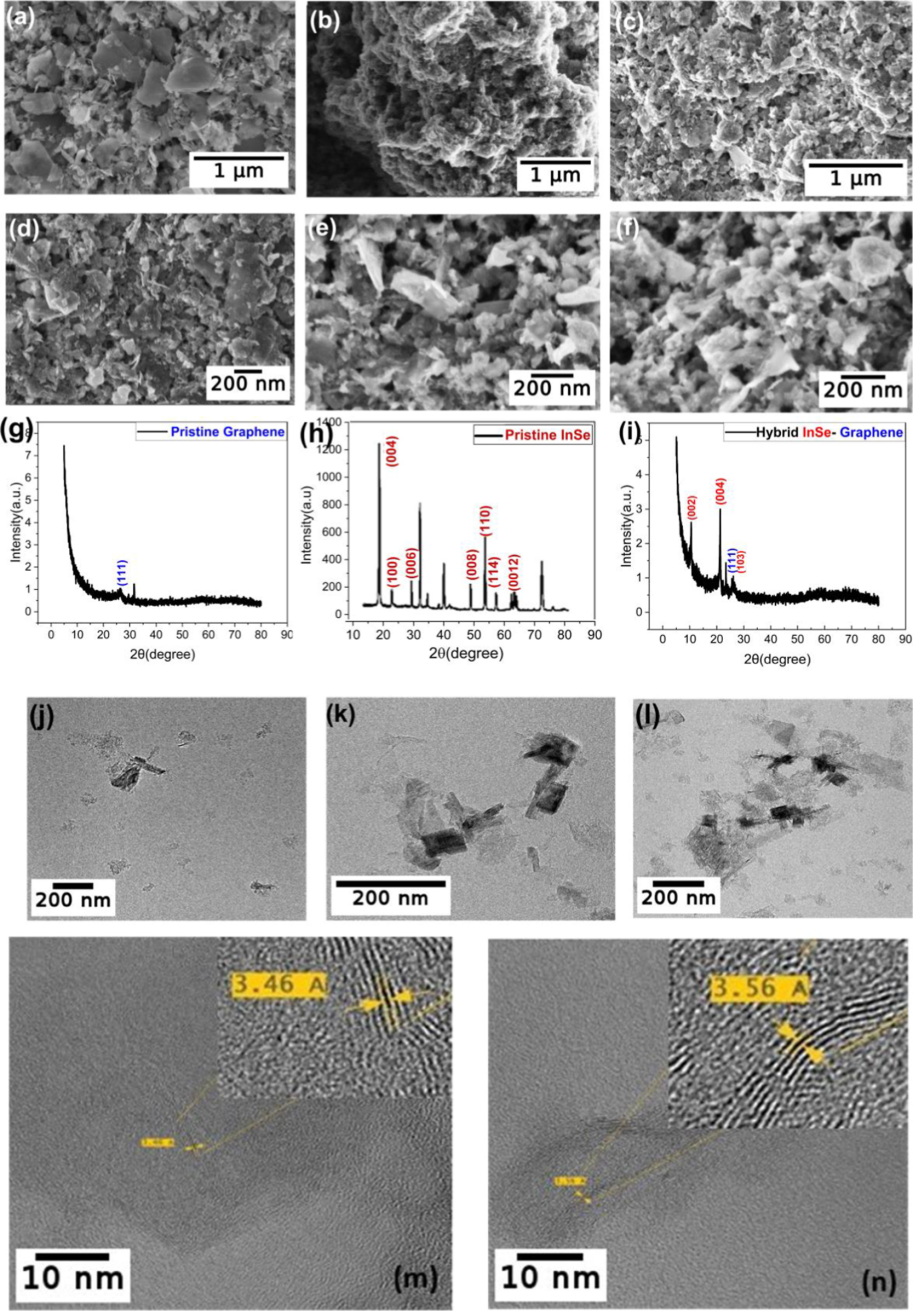
FESEM Images of (a) pristine InSe; (b) pristine graphene; (c) hybrid InSe-graphene; (d) hybrid T; (e) hybrid M; and (f) hybrid B. XRD diffractograms of (g) pristine InSe; (h) pristine graphene; and (i) hybrid InSe-graphene. HRTEM results of nanoflakes from (j) hybrid T; (k) hybrid M; and (l) hybrid B. HRTEM of hybrid InSe-graphene with (m) zoomed-in InSe layers and (n) zoomed-in graphene layers.

The general hexagonal form of graphene does not change, but long-term exposure to shock waves and shear stress during sonication causes its disarray and increased fragmentation at the edges.^52^ Long ultrasonication is also used to produce graphene oxide from graphene. According to the literature, when sonication lasts more than 4 h, the oxidation of graphene increases.^53^ To confirm the increase in oxygen moieties on graphene, we performed FESEM–EDX elemental mapping of the hybrid sensor and discovered a C/O ratio of 1.5:1 ^51^ (see Figure S3 in Supporting Information). Oxidized graphene is composed of sp^2^ and sp^3^ hybridization, in which oxygen-containing functional groups are bonded to the lattice and the interlayer distance is increased. In our exfoliated materials, oxygen content has increased. This oxidation process is discussed further via a Raman study below. The formation of oxidized graphene during the exfoliation process brings about improved gas-sensing characteristics by increasing the number of active sites available for the interaction with gas molecules.^54–58^

### XRD Analysis

XRD was used to study the crystalline structures of the sensing films. The XRD diffractograms obtained from the pristine InSe, pristine graphene, and hybrid InSe-graphene are displayed in Figure 2g–i, covering the range of 2θ = 5° to 80°. The measured diffraction peaks correspond to the *P*6_3_/*mmc* space group hexagonal phase of InSe (ICDD card number: 34-1431), with lattice constants *a* = 0.400 and *c* = 1.664 nm. For the pristine InSe sample, the peaks (004), (100), (006), (008), (110), (114), and (0012) refer to 2θ = 21.29°, 25.68°, 32.25°, 43.47°, 43.30°, 50.52°, and 67.57°, respectively. The nonspecified peaks in pristine InSe are identified as alumina corundum and correspond to the substrate. Graphene diffraction peaks correspond to the rhombohedral phase (*R*3̅*m* space group) with a lattice constant of 0.36 nm (ICDD card number: 75-2078). For the pristine graphene sample, the peak at 2θ = 26.61° corresponds to graphene (111). The principal diffraction peaks for the hybrid InSe-graphene sample are indexed to the lattice planes (002) at 10.62° and (004) at 21.29° corresponding to InSe. The graphene (111) peak can also be observed in the hybrid InSe-graphene sample. The presence of peaks from graphene and InSe confirms the hybridization of InSe-graphene.

### HRTEM and Raman Studies

In addition to the morphological data from FESEM, the hybrid InSe-graphene sample was also studied via TEM for a better understanding of the thin layers. A proper sectionwise comparison of nanosheets can be observed from T to B in hybrid InSe-graphene sensors. The number and size of the nanosheets increased moving toward the bottom of the suspension, which was also evident in the FESEM results. A graphene-hybridized crystalline layered structure of InSe is also revealed by the analysis of the HRTEM data, as illustrated in Figure 2j–l. As illustrated in Figure 2m,n, we confirmed the interplanar distance with *d* spacing equal to 0.34 nm, which corresponds to the (101) plane of InSe (ICDD card number: 34-1431). Graphene nanosheets have a computed *d* spacing of 0.35 nm, which is equivalent to the (111) plane of graphene oxide (ICDD card number: 75-2078). It is observed that graphene transformed into oxidized graphene after being sonicated for 8 h.^53,59^ This may be the result of the smaller graphene flakes oxidizing more due to the sonication process, which attaches oxygen functionals to the graphene edges and surface.^60,61^ Raman characterizations were also performed for the hybrid InSe-graphene material employing a Renishaw inVia instrument equipped with a 633 nm, argon-Novatech, 25 mW laser. It was found that there were 3 visible peaks of graphene in the hybrid material, namely, G, D, and 2D, which fall on 1327.20, 1573.98, and 2639.45 cm^–1^, respectively. The presence of graphene is evident in Raman peaks, but InSe peaks are not visible (see Figure S4 in the Supporting Information). The presence of the D band in Raman peaks indicates the presence of defects in the graphene lattice and the G band indicates the stretching of the C–C bond.^62^ Additionally, Raman spectra were also recorded for as-received, commercially available graphene nanoplatelets before and after the exfoliation process (see Figure S5 in the Supporting Information). These results show that both as-received graphene nanoplatelets and exfoliated graphene nanoplatelets display G and D peaks. While the G peak is indicative of the sp^2^ hybridization in graphitic carbon, the D peak accounts for the defects induced on the hexagonal sheet of carbon. The ratio of the peak intensities *I*_D_/*I*_G_ can be used to characterize the level of disorder in graphene.^63^ This ratio is slightly higher for exfoliated graphene nanoflakes (1.35) than for as-received graphene nanoflakes (1.16), thus confirming that the exfoliation process results in more disordered and defective graphene. Considering that exfoliated graphene samples are exposed to the environment, it can be concluded that exfoliated graphene shows a higher amount of oxygen species than as-received graphene.^64^ This is in good agreement with the previously discussed EDX results.

### PL Spectra

Figure 3 shows the PL spectra of the hybrid graphene-InSe and pristine InSe samples. The shoulder observed at around 1.5 to 2 eV is related to the bulk InSe bandgap, and the broad emission band observed at around 2 to 3 eV is related to the single layer InSe bandgap.^29^ We observed a higher emission intensity from the hybrid InSe-graphene compared to the pristine InSe; see Figure 3a. This is because the addition of graphene to InSe makes the photon emission more efficient. Graphene allows electrons to flow quicker, making the hybrid InSe-graphene more conductive, and with higher emission intensity due to the higher recombination of electrons.^65^ Moreover, the maximum of the broad and intense defect emission peak is shifted from 2.25 to 2.40 eV for the pristine InSe and hybrid InSe-graphene, respectively; see Figure 3b. This shift of the PL peak is related to the quantity of single layers introduced by the hybrid InSe-graphene. The higher emission intensity from the hybrid InSe-graphene compared to the pristine InSe is correlated with the gas-sensing performance. In contrast to what was observed for hybrid InSe-graphene samples, it was not possible to record a chemoresistive response toward NO_2_ in pure InSe. Higher PL emission intensities correlate with higher charge carrier recombination and increased charge carrier mobility, bringing about an increase in conductivity for the hybrid nanomaterial and an enhancement in its sensing response toward NO_2_.

**Figure 3:**
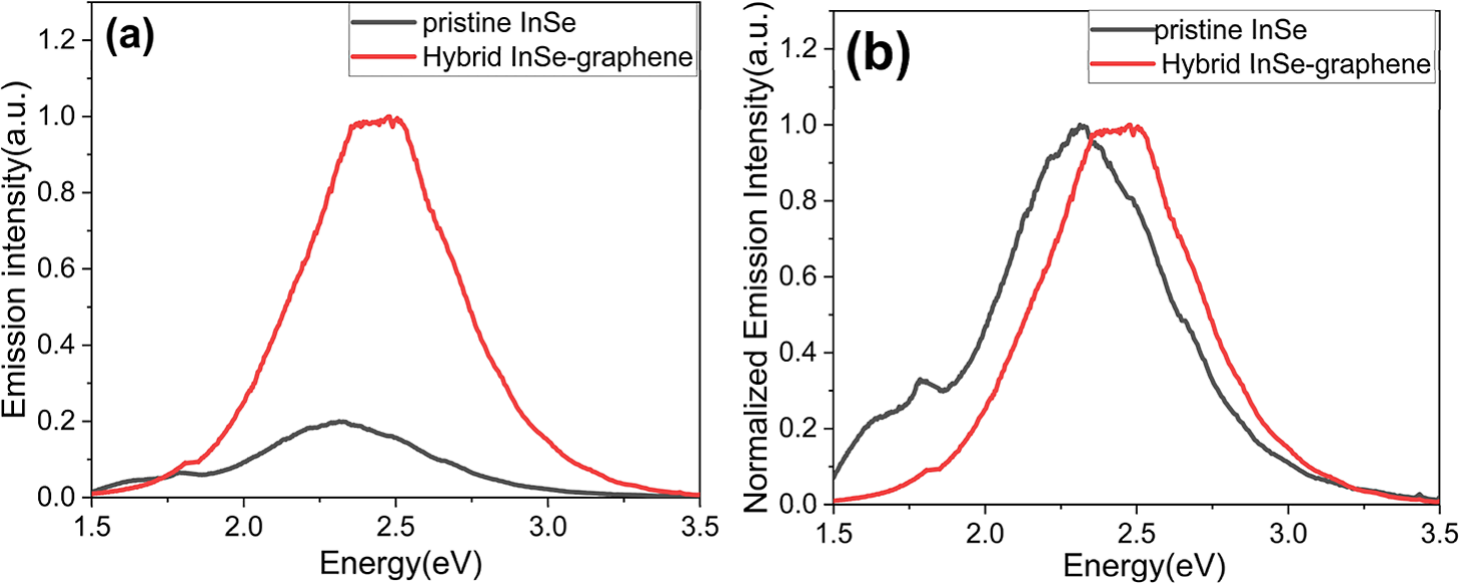
(a) PL spectra of hybrid InSe-graphene and pristine InSe. (b) Normalized emission intensity spectra for comparison between hybrid InSe-graphene and pristine InSe.

### Gas-Sensing Results

#### Comparison of Hybrid InSe-Graphene with Pristine Graphene Sensors

The sonicated solutions were divided into the 3 sections, designated hybrid T, hybrid M, and hybrid B. Each one of these solutions was employed to obtain distinct sensors to investigate the effect of dispersion sizes in the gas-sensing performance. Additionally, pristine graphene and InSe sensors were produced and tested, as well. Sensors were tested simultaneously at room temperature (RT). Sensor response (%) was defined as shown in eq 1 for reducing gases, and as given in eq 2 for oxidizing gases,

1 $$R\;\%=\frac{R_{gas}-R_{air}}{R_{air}}\times 100$$

2 $$R\;\%=\frac{R_{air}-R_{gas}}{R_{air}}\times 100$$

where *R*_air_ and *R*_gas_ are the real-time resistances of the sensor exposed to air and to the analyte, respectively. The responses for 1 ppm of NO_2_ were 1.17% for the pristine graphene sensor, 2.36% for the hybrid T sensor, 2.15% for the hybrid M sensor, and 2.70% for the hybrid B sensor. The pristine InSe sensor was very resistive (baseline resistance of few giga ohms) and no response to NO_2_ was recorded. Therefore, the pristine InSe sensor was not investigated further. In contrast, sonicated graphene and hybrid sensors exhibited low resistance values, in the range of a few hundred ohms. These values indicate that the degree to which graphene gets oxidized during the sonication process is moderate as the conductivity of the material remains quite high. Our previous results show that the resistance of GO films of similar thickness on identical electrodes as the ones used here is in the range of tens to hundreds of kΩ.^66^ The gas-sensing results showed that the responses of hybrid InSe-graphene sensors are more than two times higher than the response of pure graphene. The hybrid InSe-graphene films comprise many p-type/p-type heterostructures in which InSe plays a role in the reception function toward NO_2_ and graphene (characterized by a high carrier mobility) helps lowering the resistance of the film and provides a path for the charge carriers generated upon gas/film interaction to reach the electrodes. Second, we compared the 3 different hybrid InSe-graphene sensors (i.e., T, M, and B) to understand better the effect of selecting the materials from each of the three sections of the centrifuge-dispersed nanosheets. These results are summarized in Figure 4. It was found that the responses of sensor hybrid-B were the highest toward 1 ppm of NO_2_ gas. This corresponds to the bottom section of dispersion. The bottom layer has more exfoliated layers than the top and middle layers because when the exfoliated material is centrifuged, the larger nanosheets tend to accumulate more toward the bottom suspension, while the thinner flakes or particles rise to the top.^36,67^ Furthermore, because the number of layers in the B section is higher, additional defects are introduced into the nanosheets such as structural defects, grain boundaries, and vacancies. Compared to the latter, this allows for the achievement of more active sites. These variables may be the cause of a higher drop in resistance and increase in response compared to the other sensors.

**Figure 4:**
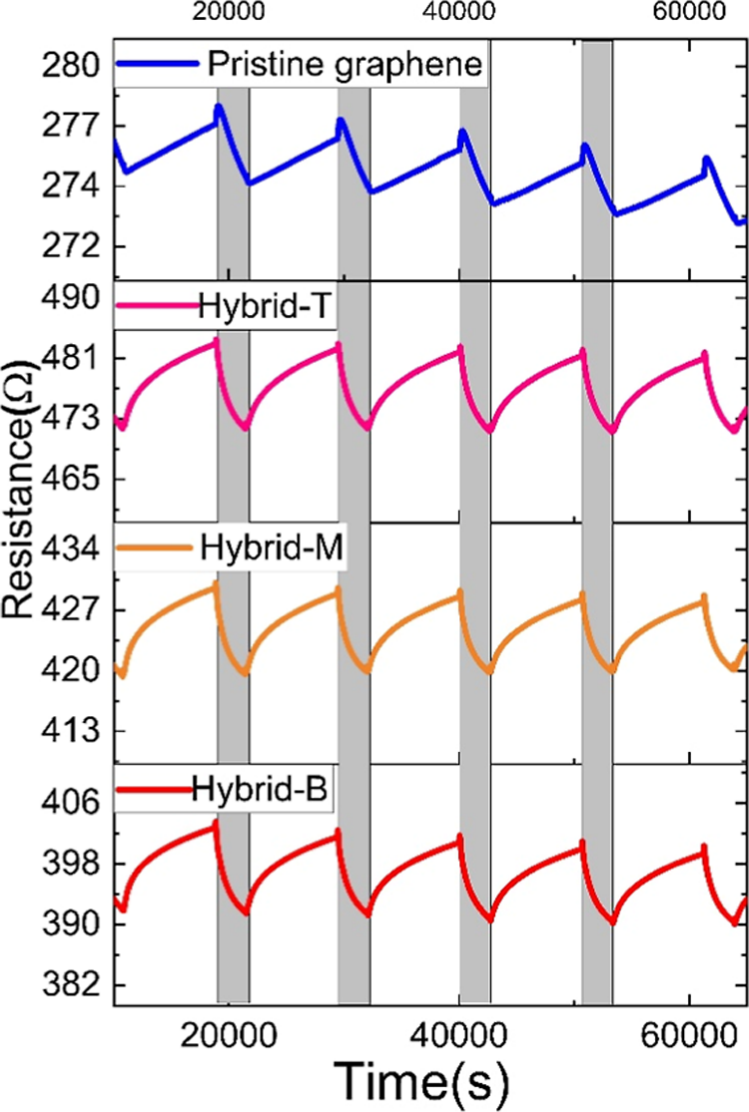
Different sensors comparison based on dispersion of layers with response to 1 ppm of NO_2_ at RT.

### Operating Temperature Study

We analyzed the impact of the operating temperature on the sensing behavior of hybrid InSe-graphene sensors by operating them from room temperature to 200 °C under dry conditions. We found that, for a temperature range comprised between RT and 150 °C, the sensor response increased with temperature because higher temperatures enhanced the rate of gas molecule adsorption and reaction on the surface, which is a crucial stage in the gas-sensing process. Temperatures above RT can also accelerate the desorption of previously adsorbed gas molecules, allowing the sensor to recover faster between gas exposure cycles.^68^

However, at temperatures above 150 °C, the response of both the pristine graphene sensor and the hybrid InSe-graphene sensor decreased and became noisy due to increased desorption rate (see Figure S6 in the Supporting Information). Additionally, high temperatures can cause structural changes in InSe, leading to phase transitions or degradation (e.g., oxidation), affecting its sensing characteristics and introducing noise and instability into sensor responses.^69^ Similarly, thermal instability in graphene can cause changes in its electrical properties and conductivity, affecting its gas-sensing capabilities. Thus, the optimal operating temperature for the hybrid InSe-graphene sensor when operated in a dry environment was found to be 150 °C. Figure 5 summarizes these results. The error bars indicate the uncertainty associated with the measurements. For every operating temperature studied, 5 replicate, independent measurements were performed per gas concentration. The error remains below 10% for all temperatures and concentrations tested. We also tested the sensors at RT with higher NO_2_ concentrations, and their response did not get saturated, indicating that they can function effectively at high NO_2_ concentrations as well (see Figure S7 in the Supporting Information).

**Figure 5:**
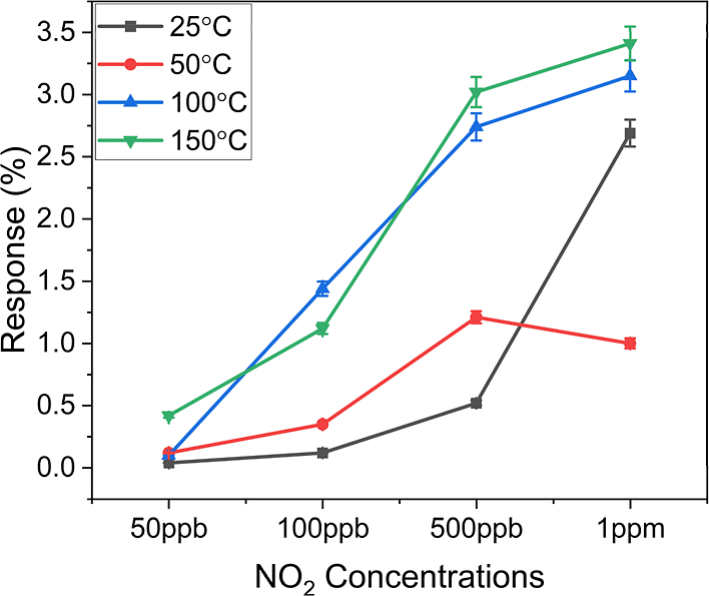
Hybrid InSe-graphene sensor responses to NO_2_ at different concentrations for varied operation temperatures.

### Effect of Humidity

Figure 6 summarizes the effect of humidity on the sensing behavior of the hybrid InSe-graphene sensor. Figure 6a shows the responses toward repeated exposures of 1 ppm of NO_2_ while under humid (50% relative humidity at 25 °C) or dry conditions. On the other hand, Figure 6b analyzes the response to NO_2_ under humid conditions for different operating temperatures. The water-mediated enhanced response to NO_2_ observed here has been reported previously in metal oxides and in transition metal dichalcogenides supported on carbon nanomaterials.^70–72^ Exposure to humidity results in an increase both in isolated and bridging hydroxyls at the surface, which play a role in favoring the adsorption of NO_2_ and the appearance of nitrites (NO_2_^–^).^62,73^ At higher operating temperatures (>200 °C), the oxidation of nitrites into nitrates (NO_3_^–^) has been reported as well.^59^ While response intensity remains quite stable in the RT to 200 °C range, a significant increase is observed at the operating temperature of 250 °C.

**Figure 6:**
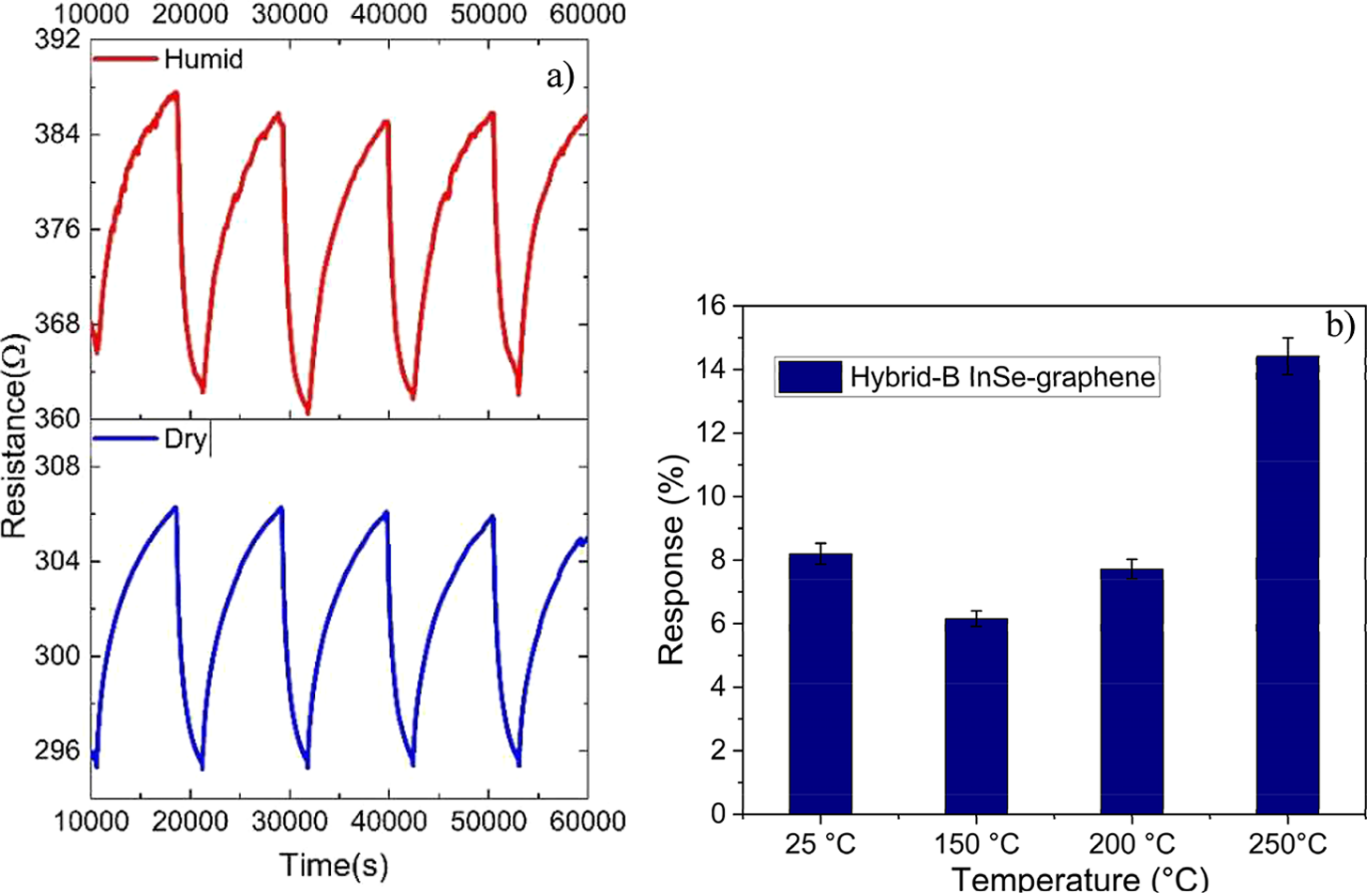
(a) Comparison of hybrid B InSe-graphene sensor response under dry and humid conditions. Sensor operated at 150 °C. (b) Hybrid-B sensor responses at different operating temperatures under humid conditions.

The sensor could now be tested at higher operating temperatures of 200–250 °C due to the improved performance observed under humid conditions. However, operating temperatures above 250 °C may damage the sensor by degrading the gas-sensitive nanomaterials and were not explored. Sensor response increased from 6.16% to 14.42% when the operating temperature was raised in the 150–250 °C range, under humid conditions (50% RH). We considered the response and recovery time of sensors in humid environments. As sensors decrease their resistance in the presence of NO_2_, we have defined response and recovery times as follows. Response time is the time it takes for a sensor to reach a resistance 10% above its steady-state resistance value when it is exposed to a step change in the concentration of a given target gas. Recovery time is the time it takes for a sensor to return to 90% of its baseline value when suddenly exposed to pure air. Response and recovery times of the sensor are reported in the Supporting Information (see Section S1).

### Selectivity Tests

The sensor was tested for selectivity with different interfering species, including CO (50 ppm), benzene (1 ppm), and CO_2_ (100 ppm) apart from NO_2_. Results show that the hybrid sensor has a significantly low cross-sensitivity to CO, benzene, and CO_2_. The response for CO, CO_2_, and benzene was less than 1% (see Figure 7). It can be derived that the hybrid InSe-graphene sensor has excellent NO_2_ selectivity. Table S7 (see the Supporting Information) enables comparing the performance of the hybrid InSe-graphene sensor against one of the previously reported chemoresistive sensors employing InSe.

**Figure 7:**
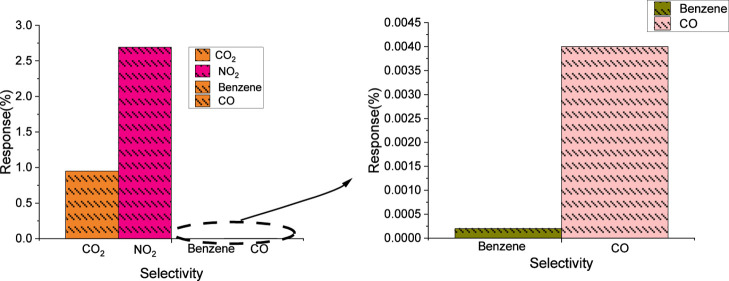
Selectivity test for different gases. Sensor operated at room temperature under dry conditions.

### Sensing Mechanism

Chemoresistive gas sensors use electrical resistance modulation as their detecting method. This modulation is caused by chemical reactions between the target gas and the sensor surface, which interact with one another.^74^ NO_2_ is an oxidizing species (electron acceptor), and thus, upon adsorption, electronic charge is transferred from the gas sensitive film toward the molecule. The transferred electrons cause the electrical resistance of the film to change. Depending on whether the sensitive material is an n-type or p-type semiconductor, resistance increases or decreases when NO_2_ is adsorbed.^75,76^

The gas-sensing mechanism of transition metal chalcogenides is primarily based on charge transfer between the analyte gas molecule and the gas detecting layer.^77^ In the case of InSe, the sensitive layer interacts with the NO_2_ gas molecules on the surface, resulting in decreased resistance. Because NO_2_ acts as an electron-withdrawing gas, it removes electrons from InSe, increasing the concentration of holes.^27^

As reported by Zhang and co-workers,^27^ the sensing mechanism of pure InSe gas sensors mainly comprises the electron transfer caused by surface redox reactions and changes in carrier concentration, in which the following reactions take place.

3 $$O_{2}+e^{-}\to O_{2}^{-}\;\left(ad\right)$$

4 $${NO}_{2}\left(gas\right)+e^{-}\to {NO}_{2}^{-}\;\left(ad\right)$$

Given the exposure of the sensor to the environment, negatively charged molecular oxygen species are adsorbed on the InSe. Upon exposure to NO_2_, computational chemistry studies indicate that this molecule also adsorbs on the surface of InSe resulting in a transfer of a fraction of electronic charge from the InSe toward the adsorbed molecule. In our hybrid InSe/graphene material, the direct interaction between nitrogen dioxide and graphene flakes surely takes place. Indeed, sensors employing exclusively exfoliated graphene remain responsive to nitrogen dioxide. However, sensors employing the hybrid nanomaterial show a response intensity more than 2-fold higher than that employing exfoliated graphene only. Therefore, the direct interaction between InSe and NO_2_ described above is instrumental for achieving this response enhancement.

The transient resistance graphs demonstrate a decrease in resistance upon exposure to NO_2_, showing that the hybrid InSe-graphene behaves as a p-type semiconductor (holes operate as the majority carriers). The addition of graphene to InSe increases the number of adsorption sites available, resulting in higher response to NO_2_.^78^ The interaction between gas molecules is largely influenced by the presence of active sites, surface edges, and the number of functional groups.^79,80^ Additionally, as we have discussed in earlier sections, our sensor behavior makes it evident that, in addition to offering high selectivity toward NO_2_, it also demonstrates improved responses when operated under humid conditions. The prolonged sonication process undergone by graphene to obtain a suspension of graphene nanoflakes induces the presence of oxygenated defects.^52,53,81^ This increases the number of active sites for interacting with gas molecules^54–57^ and favors the adsorption of water molecules. At the operating temperatures employed, water adsorption results in the presence of terminal and bridging hydroxyls on the surface of the hybrid nanomaterial and their role on enhancing response to NO_2_ has been reported previously.^72,73^

## Conclusions

We have showcased a highly selective hybrid NO_2_ sensor that is resilient to ambient humidity without the need for being light-activated. In humid settings, the formation of hydroxyl groups mediates the adsorption of NO_2_ leading to a noticeable increase in response. The sensor was also tested with other different gases apart from NO_2_ like CO, CO_2_, and benzene. The sensor responses to these other species were negligible (less than 1%), which confirms the high sensor selectivity for NO_2_, even if operated at room temperature. The results showed that, at 1 ppm of NO_2_ at room temperature, the hybrid sensor has a considerably higher response than the pristine graphene sensor. InSe is a p-type nanomaterial that decreases its resistivity when it interacts with an electron acceptor such as NO_2_. The addition of graphene to InSe in a hybrid InSe-graphene sensor results in an increased number of adsorption sites for the hybrid nanomaterial and an enhancement in its sensing response toward NO_2_. Additionally, the results showed that the hybrid exhibits excellent stability and selectivity in a broad range of NO_2_ concentrations and even when operated at RT. This nanomaterial shows, hence, promise for being used in real-world applications.

## Supplementary Material



## Notes

Image of an alumina substrate with platinum electrodes; FESEM images for bulk InSe; chemical mapping (microanalysis) results for a hybrid InSe-graphene sample; Raman peaks recorded on hybrid InSe-graphene; Raman spectra recorded on as-received, commercially available graphene nanoplatelets and on the same graphene nanoplatelets having undergone an exfoliation process; hybrid InSe-graphene sensor response for 1 ppm NO_2_ at 200 °C; hybrid InSe-graphene sensor response toward high concentrations of NO_2_; response and recovery times of sensor; and table enabling the comparison of sensor performance against previously reported results (PDF)
