# Supplementary material for: Highly Selective Hybrid InSe-Graphene for NO2 Gas Sensing with High Humidity Tolerance
Source: ACS Sens. 2025 Jul 1;10(7):4862–72. doi: 10.1021/acssensors.4c03521 (PMC12305650; doi:10.1021/acssensors.4c03521)
Supplement: Supplementary file 1 [file se4c03521_si_001.pdf]

Supporting Information for

Highly selective hybrid InSe-graphene for NO_2 gas sensing with high humidity tolerance

Jyayasi Sharma[1,2,3], Frank Güell[4], Mubdiul Islam Rizu[1,2,3], Dalal Fadil[1, 2,3*], Eduard Llobet[1,2,3*]

[1] *Universitat Rovira i Virgili, MINOS, School of Engineering, Avda. Països Catalans 26, 43007 Tarragona, Spain*
[2] *IU-RESCAT, Research Institute in Sustainability, Climatic Change and Energy Transition, Universitat Rovira i Virgili, Joanot Martorell 15, 43480 Vila-seca, Spain*
[3] *TecnATox - Centre for Environmental, Food and Toxicological Technology, Universitat Rovira i Virgili, Avda. Països Catalans 26, 43007 Tarragona, Spain*
[4]*Universitat de Barcelona, Catalan Photonics for Energy (ENFOCAT),08028 Barcelona, Catalunya, Spain*
**corresponding authors: dalal.fadil@urv.cat; eduard.llobet@urv.cat*

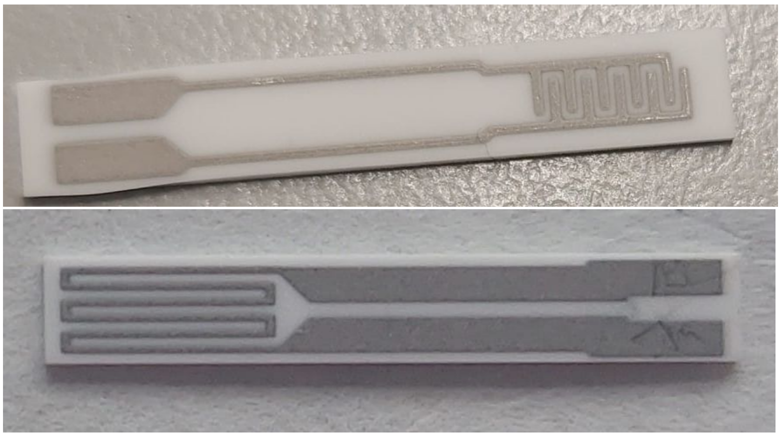

Figure S1. Image of an alumina substrate with platinum electrodes interdigitated with 300 µm gap and meander Pt heater on the back.

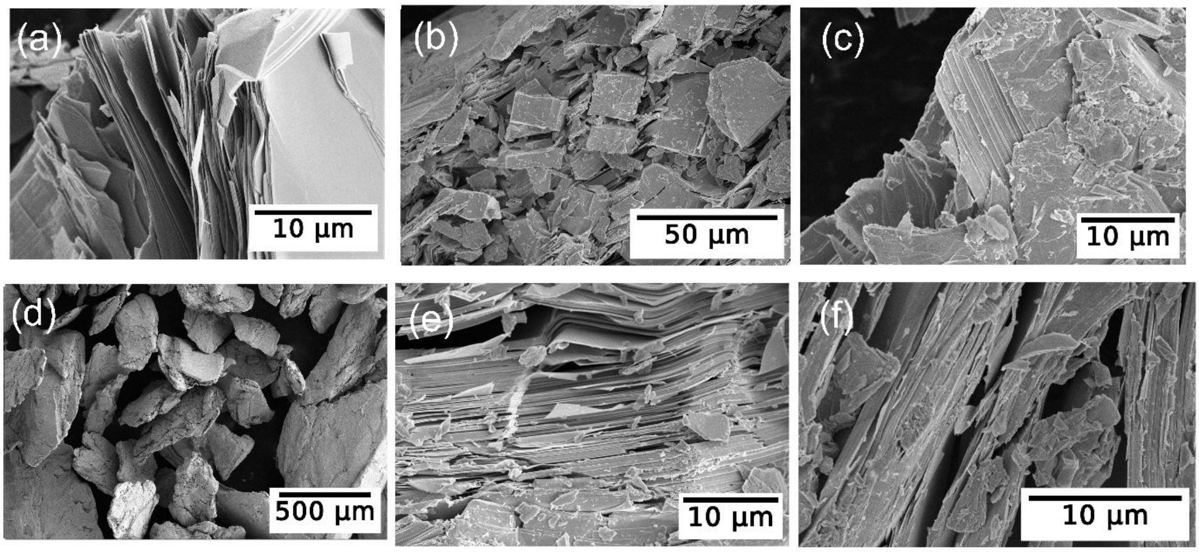

Figure S2. FESEM images for bulk InSe

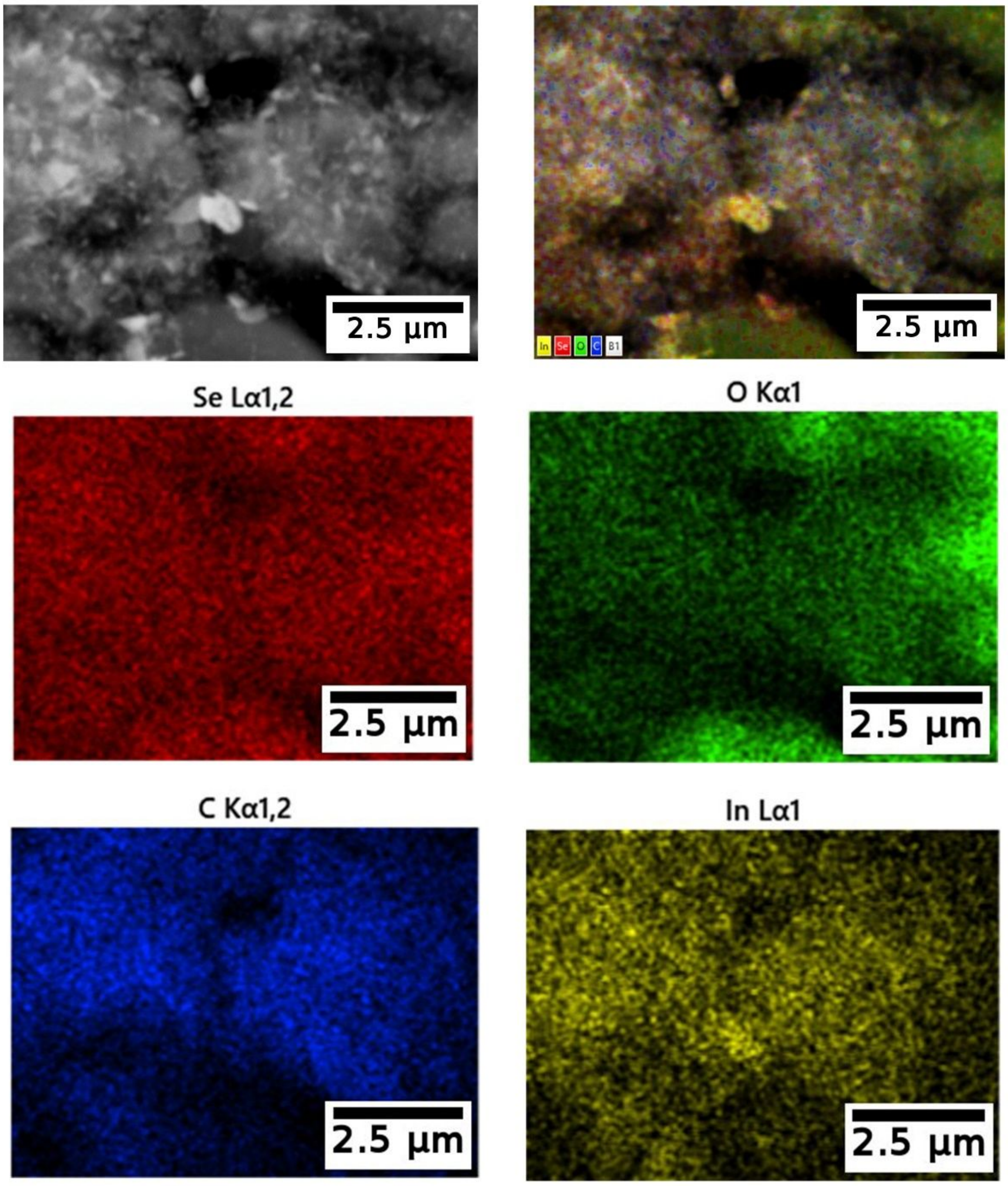

Figure S3. Chemical mapping (microanalysis) results for a hybrid InSe- graphene sample.

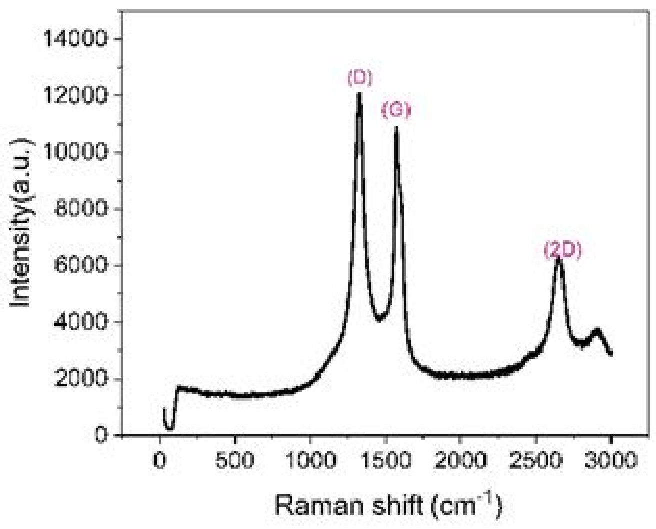

Figure S4. Raman peaks recorded on a hybrid InSe-graphene nanomaterial.

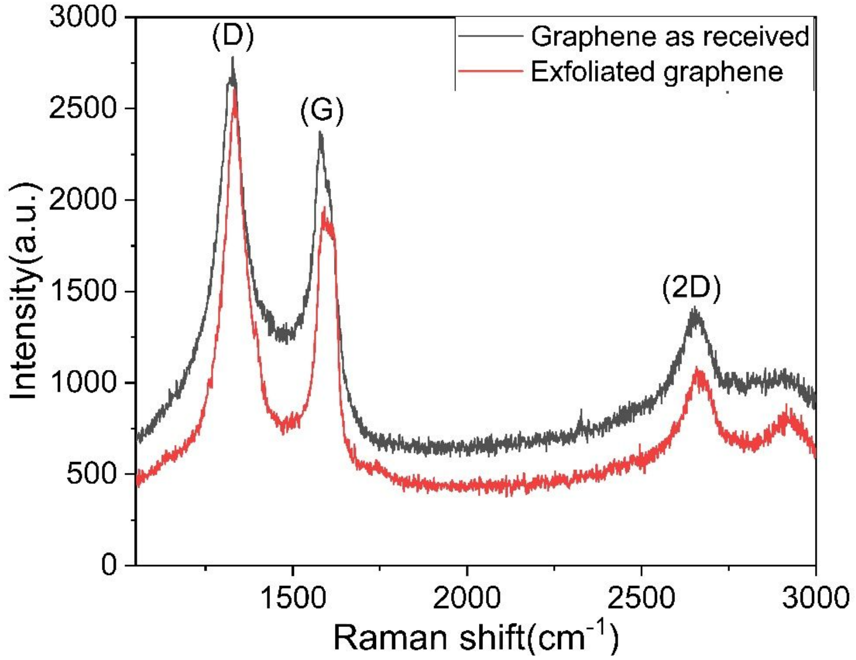

Figure S5. Raman spectra recorded on as-received, commercially available graphene nanoplatelets and on the same graphene nanoplatelets having undergone an exfoliation process.

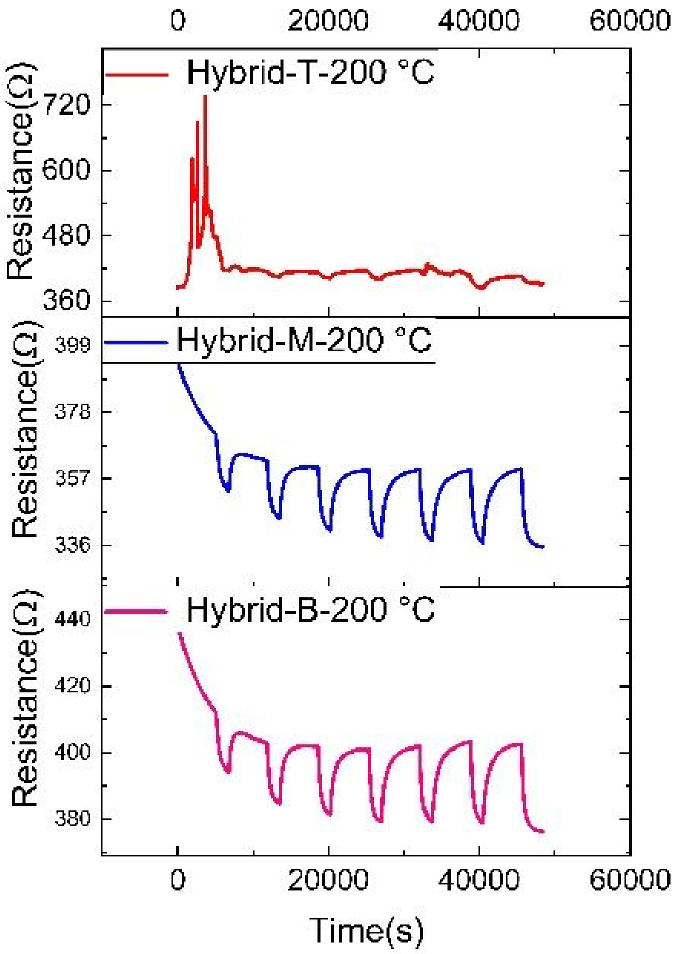

Figure S6. Hybrid InSe- graphene sensor response for 1 ppm -NO_2 at 200℃ for the study of the optimum operating temperature.

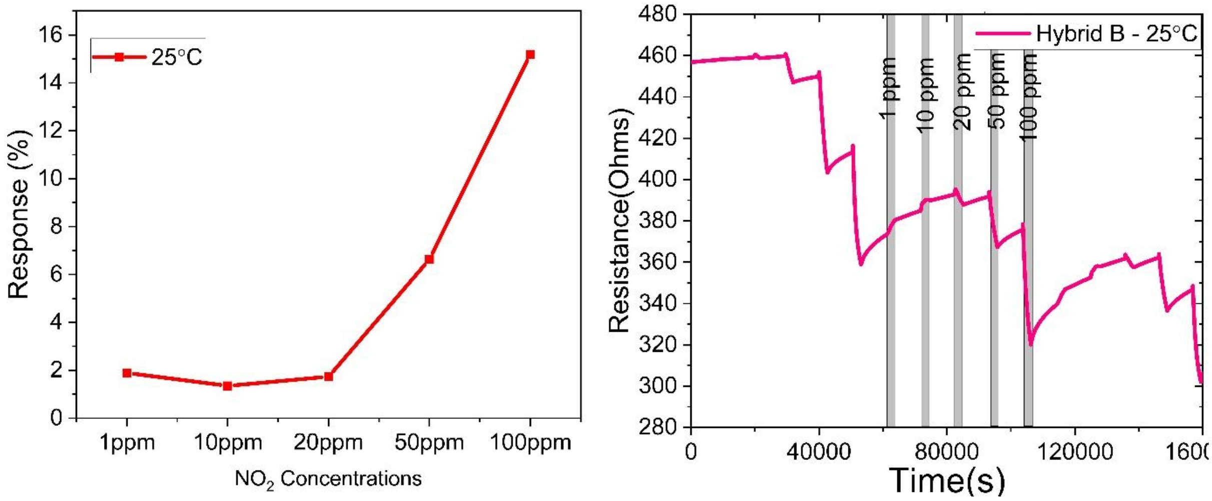

Figure S7. Hybrid InSe-graphene sensor response towards high concentrations of NO_2.

Section S1. Response and Recovery Time of sensor-

We have taken into account the response and recovery time for 150 ℃ and 250 ℃, both in the presence of 50 % R.H. The response and recovery time for 150 ℃ are as follows (363.3 s / 406.8 s) and for 250 ℃, response and recovery time are as follows (193.2 s / 462 s). This difference in time can be attributed to the fact that the response time is negatively correlated with temperature and hydrogen concentration [1]. With the increment of operating temperature, the response time has shortened significantly, while recovery time does not show much of a difference.

Table S1. Literature Review

Sensing Material	Tested range/ LoD	Response%/ Concentration	Working state	Humidity effect	Cross sensitivity	Ref.
1.InSe nanosheets/ Au IDEs	0.05-5 ppm/ 0.98 ppb	190% / 1 ppm NO_2<br><br>402% / 1 ppm NO_2	3.2 mW/ cm^2, UV light, air, 30℃<br><br>0.8 mW/ cm^2, UV light, air, 30℃ Flow*: 1000 sccm	Response degraded at 60% R.H.	Cross-responsive to H_2S	2
2. $PdSe_2$/ InSe heterojunction	0.1- 20 ppm/ 100 ppb	48.4% / 5 ppm NO_2<br><br>57.6%/ 10 ppm NO_2	365 nm, UV light, RT Flow: N/A	Slight decrease in response 85% R.H.	Good selectivity to NO_2	3
3. In-Se mixed oxides	0.5-50 ppm/ 1 ppm	8 / 3 ppm NO	150℃ Flow: N/A	Not tested	Sensitive to NO, no response to NO_2	4
4. InSe nanosheets	0.1- 20 ppm/ 10 ppb	2.75 / 10 ppm NO_2	395 nm, UV light, RT Flow: N/A	Degraded response at 75% R.H.	Cross-sensitive to triethylamine	5
5. CTAB intercalated InSe nanoscrolls	0.1- 10 ppm/0.43 ppb	4890%/ 10 ppm NO_2	620 nm, visible light, RT	Response degraded at 60% R.H.	Small cross-sensitivity to H_2 and CO	6

			Flow: 1000 sccm			
6. InSe-graphene	0.05- 100 ppm/ <50 ppb	8%/ 1 ppm NO_2<br><br>14.42%/ 1 ppm	25ºC 50% R.H. 250℃, 50% R.H. Flow: 100 sccm	Response almost doubled under 50% R.H.	Good selectivity to NO_2	This work.

*High flows result in higher responsiveness.

References

(1) Liu, Q.; Yao, J.; Wang, Y.; Sun, Y.; Ding, G. Temperature Dependent Response/Recovery Characteristics of Pd/Ni Thin Film Based Hydrogen Sensor. *Sensors Actuators, B Chem.* **2019**, *290* (April), 544–550. https://doi.org/10.1016/j.snb.2019.04.024.

(2) Zhang, L.; Li, Z.; Liu, J.; Peng, Z.; Zhou, J.; Zhang, H.; Li, Y. Optoelectronic Gas Sensor Based on Few-Layered InSe Nanosheets for NO2detection with Ultrahigh Antihumidity Ability. *Anal. Chem.* **2020**, *92* (16), 11277–11287. https://doi.org/10.1021/acs.analchem.0c01941.

(3) Fan, J. Le; Hu, X. F.; Qin, W. W.; Liu, Z. Y.; Liu, Y. S.; Gao, S. J.; Tan, L. P.; Yang, J. L.; Luo, L. B.; Zhang, W. UV-Light-Assisted Gas Sensor Based on PdSe2/InSe Heterojunction for Ppb-Level NO2 Sensing at Room Temperature. *Nanoscale* **2022**, *14* (36). https://doi.org/10.1039/d2nr03881a.

(4) Serra, A.; Micocci, G.; Di Giulio, M.; Manno, D.; Tepore, A. Thermal Deposition and Characterisation of In-Se Mixed Oxides Thin Films for NO Gas Sensing Applications. *Sensors Actuators, B Chem.* **1999**, *58* (1–3), 356–359. https://doi.org/10.1016/S0925-4005(99)00096-9.

(5) Zheng, W.; Yang, C.; Li, Z.; Xie, J.; Lou, C.; Lei, G.; Liu, X.; Zhang, J. Indium Selenide Nanosheets for Photoelectrical NO2 Sensor with Ultra Sensitivity and Full Recovery at Room Temperature. *Sensors Actuators, B Chem.* **2021**, *329*. https://doi.org/10.1016/j.snb.2020.129127.

(6) Zhang, L.; Hao, Q.; Liu, J.; Zhou, J.; Zhang, W.; Li, Y. Rolling up of 2D Nanosheets into 1D Nanoscrolls: Visible-Light-Activated Chemiresistors Based on Surface Modified Indium Selenide with Enhanced Sensitivity and Stability. *Chem. Eng. J.* **2022**, *446* (P2), 136937. https://doi.org/10.1016/j.cej.2022.136937.
